# Clinical correlation between erectile function and ejaculatory function in the Czech male population

**DOI:** 10.1371/journal.pone.0199588

**Published:** 2018-07-12

**Authors:** Watcharaphol Alexandre Kamnerdsiri, Jesús Eugenio Rodríguez Martinez, Christopher Fox, Petr Weiss

**Affiliations:** 1 Department of Psychiatry, First Faculty of Medicine, Charles University, Prague, Czech Republic; 2 Murcian Institute of Sexology, Murcia, Spain; 3 Westmead Clinical School, Faculty of Medicine and Health, University of Sydney, Sydney, Australia; 4 Institute of Sexology, First Faculty of Medicine, Charles University, Prague, Czech Republic; Northwestern University, UNITED STATES

## Abstract

**Introduction:**

This study explores the relationship between erectile function and ejaculatory function, to inform the clinical psychosexological and sexual medicine practice treatment protocols.

**Materials and methods:**

A total of 1,004 Czech males aged between 15 and 84 years (*m* = 42.8 yrs; *sd* = 17.6 years) completed a sexual behavior questionnaire. A cross-sectional design was adopted. Erectile function was measured with the International Index of Erectile Function (IIEF-5) and ejaculatory function measured using self-report intravaginal ejaculation latency time and the Index of Premature Ejaculation (IPE). Linear regression analyses were used to explore the relationships between premature ejaculation and erectile dysfunction.

**Results:**

The sample mean self-reported intravaginal ejaculatory latency time was 9.34 minutes. The overall mean on the IPE was 19.44 (*sd* = 2.368). The *Control* domain mean was 81.13 (*sd* = 17.22); *Sexual Satisfaction* domain mean 78.60 (*sd* = 20.59); and the *Distress* domain mean was 86.86 (*sd* = 18.32). The mean score on the IIEF-5 was 19.28 (*sd* = 2.53). The results indicate a relationship between premature ejaculation and erectile dysfunction. With age significantly associated with all measures.

**Conclusions:**

Higher levels of erectile function are associated with a better control and sexual satisfaction, and less distress about ejaculation. This association supports the consideration of this relationship in the development of new clinical practice guidelines for erectile dysfunction and premature ejaculation.

## Introduction

Sexual dysfunction is characterized by distress with changes during any stage of the sexual response cycle. Sexual dysfunctions can affect men and women at any time of their lives and impact on perception of sexual satisfaction [1]. In men, erectile dysfunction (ED) and premature ejaculation (PE) are the two main complaints [2].

Erectile dysfunction is defined as the persistent inability to attain and/or maintain an erection to permit satisfactory sexual performance [2]. It is estimated that it affects up to 52% of men, from which 5% to 20% experience moderate to severe symptoms [2,3]. Furthermore, it is believed that erectile function can be influenced by psychological disorders [4], including depression [5], anxiety [6,7] and panic attacks [8].

Premature ejaculation (PE) is defined as a persistent or recurrent ejaculation with minimal sexual stimulation before, on, or shortly after penetration and before the person desires ejaculation [9]. It is considered the most common sexual dysfunction in men, with prevalence reported from 3% to 30% [10–12] Men with PE also report reduced or absent perceived ejaculatory control, and the presence of negative personal consequences [13]. Premature ejaculation is strongly associated with performance anxiety [14].

One in three men with ED, also experience PE [15]. Jannini, Lombardo and Lenzi explain the ED-PE interaction as a result of the man’s attempt to control his ejaculation results in a reduction in his excitement level (which can result in ED). The resultant ED leads to a greater focus on arousal (and excitement) to encourage the erection to return with this increased excitation leading to PE[16].

Although erection and ejaculation occur at different phases of a man’s sexual response there are commonalities. Both processes are associated with pelvic floor muscles and good function contributes to better erections and ejaculatory control [17]. Psychological distress, anxiety (including performance anxiety) and depression are associated with erectile dysfunction and premature ejaculation [6,7,8,18]. The comorbidity of EF and PE is connected through a number of physiological and psychological processes and variables.

Good sexual function has a positive impact on people’s sexual and psychological wellbeing [19–21]. A healthy sex life has been reported to contribute to better physiological and psychological function for men and women including better sex-life satisfaction, higher levels of relationship satisfaction, increased mental health and greater satisfaction with general life [22,23]. Healthy erections (erectile quality and duration) has been reported to be associated with better outcomes for female partners and include improved body image [24], a higher levels of relationship satisfaction [25] along with higher probability of orgasm and sexual satisfaction.

Pleasure is considered one of the main objectives of human sexual expression. Men’s perception of increased duration of sexual play leads to increased enjoyment for him and his partner [16]. With the desire for increased pleasure, ejaculation time has become central to a couple’s perception of sexual and relationship satisfaction. Althof and colleagues, along with Giulano and colleagues reported better control over ejaculation has a significant positive effect on perceived satisfaction with sexual intercourse and an inverse effect on the level of personal distress associated with rapid ejaculation [26,27].

This research explores the relationship between premature ejaculation and erectile dysfunction [28]. The specific determinants and underlying factors linking ED and PE have yet to be clearly identified [28]. Therefore, the aim of this study is to analyze the relationship between erectile function and three dimensions of ejaculatory function: control over ejaculation, satisfaction with ejaculation, and distress levels caused by early or rapid ejaculation in a sample of Czech men. The research outcomes will contribute to the development of a psychosexological treatment protocol for the PE-ED as co-morbid conditions.

## Materials and methods

### Sample

The sample of 1,004 Czech men aged between 15 and 84 years had a mean age of 42.8 years (*sd* = 17.6). Age categories are reported in Table 1. Approximately two-thirds reported not being in a relationship (single = 38.1%; divorced = 13.1%; widowed = 6.2%), and 42.6% of the sample were married or in a relationship. Participants were more likely to have completed secondary studies (39.1%) or vocational training studies 30%), than university education (19.1%) or completing primary studies 11.8%). Eighty-eight per cent of the participants identified as heterosexual with 10.7% unedifying as bisexual and 1.4% as same-sex attracted. The study participation rate was 82%.

**Table 1:** Age by categories.

Age Category	Number of Participants (n = 1,004)	Per cent
**Up to 19 years**	96	9.6
**20–29 years**	173	17.2
**30–39 years**	187	18.6
**40–49 years**	139	13.8
**50–59 years**	141	14.0
**60–69 years**	164	16.3
**70–79 years**	57	5.7
**80–89**	3	0.3

### Procedure

Participants completed an anonymous survey pack. The survey instrument contained demographic questions, a sexual history, the International Index of Erectile Function Five item version (IIEF-5) and the Index of Premature Ejaculation (IPE). The completed survey pack was returned to the researchers. Ethical approval was granted by the First Faculty of Medicine of Charles University in Prague, Czech Republic.

### Instruments

The shorter, five-item version of the International Index of Erectile Function (IIEF-5) was used to measure erectile function. The full IIEF consists of 15 items and investigates five domains. The IIEF is a valid and reliable instrument developed in conjunction with the clinical trial program for sildenafil [29,30], with a high reliability in the detection of the effects of erectile dysfunction treatment [29]. The IIEF has a very high internal consistency (*α* = 0.82–0.96 [29]. The IIEF-5 is a tool that is widely used in screening erectile dysfunction due to its fast implementation. The IIEF-5 was validated with reported internal consistency with Cronbach’s alpha scores of 0.98 and 0.88 [31,32]. Lower scores indicate greater severity of ED with men who report scores under 21 are considered to be experiencing erection issues.

Ejaculatory control was assessed using the Index of Premature Ejaculation (IPE), a valid and reliable instrument with three domains: (1) evaluation of control over ejaculation, (2) satisfaction with ejaculation, and (3) distress associated with PE [27]. The IPE consists of 10 items with a very good internal reliability and very high test/retest reliability with results ranging between 0.70 and 0.90 [27]. Convergent validity was reported as excellent for all three domains (control = 0.75; *satisfaction* = 0.60; and *distress* = 0.68). Convergent validity was assessed against intravaginal ejaculatory latency time. Scores in each domain can range from 0 to 100, with higher scores indicating greater control over ejaculation, satisfaction with ejaculation, and lower distress due to ejaculation. The IPE does not have a cut-off points on each domain and is not a diagnostic tool.

## Results

Participants self-reported ever experiencing a sexual problem (*n* = 126; 12.5%) and currently experiencing a sexual problem (*n* = 133; 13.2%). Of those men who had experienced a sexual problem, 43 men reported PE (4.3%). The mean age of the experience was 20.43 years (*sd* = 6.79) with onset as young as 11 years and as old as 45 years. Forty-six men reported a past history of ED (4.6%). The mean age for this sub-sample was 41.13 years (*sd* = 18.509) with ages ranging from 15 to 72 years. Of those currently experiencing a sexual problem, 12 men reported PE (1.2%) and 75 men reported current ED issues (7.5%)

The mean self-reported intravaginal ejaculatory latency time (IELT) was 9.34 minutes (*sd* = 10.37), with 89 men reporting ILTS of less than 1 minute (8.9%), and a further 51 men reporting between one and two minutes ILTS (5.1%). The IELT estimates are reported in Table 2. Twelve men (1.2%) reported they currently experienced premature ejaculation (No = 50; missing = 942). There was no significant correlation between self-reported premature ejaculation and researcher-diagnosis (based on IELT). An independent sample *t*-test resulted in no significant differences on self-reported IELT and perception of premature ejaculation. A significant negative correlation exists between age and self-reported IELT (*r* = -.234; *p* < .001). A one-way analysis of variance was conducted to explore the impact of age on self-reported IELT. There was a statistically significant difference between age-groups: *F* (7,790) = 6.193, *p* < .001.

**Table 2:** Self-reported intravaginal latency times.

ILTS	Number of Participants (n = 1,004)	Per cent
**< 2 mins**	149	14.84
**3–10 mins**	467	46.51
**11–15 mins**	64	6.37
**16–20 mins**	54	5.38
**21–30 mins**	37	3.69
**30+ mins**	8	0.80
**Don’t know**	37	3.69
**Did not report at all**	188	18.73

### Index of premature ejaculation

The mean scores of participants on the three domains of the IPE are reported in Table 3. A statistically significant negative association was recorded between age and the control domain (*r* = -.251; *p* < .001); sexual satisfaction domain (*r* = -.259; *p* < .001); and the distress domain (*r* = -.176; *p* < .001). Analyses of variance were undertaken to explore the impact of age on each of the domains with significant differences recorded between age-groups on each domain: *control* domain (*F* (6,631) = 9.729, *p* < .001); *sexual satisfaction* domain (*F* (6,629) = 10.812, *p* < .001); and *distress* domain (*F* (6,633) = 4.444, *p* < .001).

**Table 3:** Mean scores, standard deviations and ranges for the domains of the IPE.

IPE Domain	Mean Score	Std Dev.	Range (min-max)
**Control**	81.13	17.72	6–100
**Sexual satisfaction**	78.60	20.59	6–100
**Distress**	86.86	18.32	13–100

A diagnosis of IELT of one minute or less was used to classify the presence of PE [33]. Eighty-nine men were identified and a dichotomous variable (yes/no) applied. Significant Pearson’s correlation was indicated on all three domains: *control* (*r* = .156; *p* < .001); *sexual satisfaction* (*r* = .214; *p* < .001); and *distress* (*r* = .109; *p* < .001).

### Erectile function

The mean score on the IIEF-5 was 19.28 (*sd* = 2.53). Scores ranged from nine (moderate ED) to 24 (No ED). No participants reported severe ED and 10.5% (*n* = 105) indicated no ED. Approximately, two-thirds of the sample recorded some level of ED. Table 4 presents the EEF-5 data. Seventy-five men (7.5%) self-reported current ED. There were no association between self-reports of ED and the IIEF-5 scores. There was a statistically significant negative correlation between age and ED (as measured by the IIEF-5; *r* = .260; *p* < .001). Significant mean differences exist between age groups on the IIEF-5 mean scores (*F* (6,727) = 10.950, *p* < .001).

**Table 4:** IIEF-5 scores.

IIEf-5 Range	Number of Participants (n = 1,004)	Per cent
**0–7 (Severe ED)**	0	0.0
**8–11 (Moderate ED)**	7	0.1
**12–16 (Mild-Moderate ED)**	105	10.5
**17–21 (Mild ED)**	549	54.7
**22–25 (No ED)**	105	10.5
**Missing**	238	23.7

The relationship between the presence of ED and the three domains of IPE was significant and positive for IIEF-5 scores and also using a dichotomous variable: presence of ED/no presence of ED. As perceived control and sexual satisfaction increased so did the IIEF-5 scores (less ED). The increase in distress marked lower distress as the IIEF-5 scores increased. Table 5 reports the correlations for these data.

**Table 5:** Correlations between IIEF-5, ED and the domains of the IPE.

Domain/Measure	IIEF-5	Presence of ED (Y/N)
**Control**	.473*	.157*
**Sexual satisfaction**	.400*	.100**
**Distress**	.424*	.115***

*p < .001

** *p* = .011

*** *p* = .003

### The PE-ED relationship

A significant association exists between the presence of PE and ED (*r* = 0.162; *p* < .001). Age has an influence on the presence of PE and ED, as well as on the three domains of the IPE. Partial correlations were conducted between IIEF-5 scores and the three domains of the IPE (C*ontrol*, *Satisfaction and Distress)* while controlling for age. An inspection of the zero-order correlations suggested controlling for age had little effect on the relationship. The*se* data are presented in Table 5.

A multiple regression was used to identify the role of age, control, satisfaction and distress in predicting ED. These independent variables explained 2.9% of the total variance (*F* (4,616) = 4.547, *p* = .001). Control was the only statistically significant predictor (*Beta* = .148, *p* = .003).

## Discussion

The rates of self-report PE in this sample were lower than reported in epidemiologic studies (4.3% *vs*. 20–30% [33–35]). The self-report rates for ED were at the lower-end of prevalence estimates (5% *vs*. 5%–20% [36]). Based on IELT measures (1–2 mins) slightly higher rates of PE were recorded for the sample and were in line with previous estimates of lifelong PE [37]. This was also the case in the application of the IIEF-5 scores to determine presence of ED–more men (approx. two-thirds) were recorded as experiencing some level of ED compared to their self-reported rates of ED. There is a discrepancy between the self-reported incidence rates and measured rates of PE and ED. There was no statistical support for any relationship between self-report and researcher-measured PE or ED. This discrepancy and lack of support between self-report and objective measures suggests that reliance on self-report of sexual dysfunctions may be problematic. This relationship warrants further investigation.

Premature ejaculation was found to be negatively correlated with age. Older men were more likely to report a shorter IELT. This was further supported through analysis were differences were found between age groups with PE more prevalent among older groups of men than younger groups of men. The relationship between the domains of the *Index of Premature Ejaculation* [38] and IELT were investigated. Men recorded as experiencing PE based on self-report IELT were more likely to perceive less lower levels of ejaculatory control, less sexual satisfaction and greater sexual distress. The domains were also explored in relation to age with older men perceiving less ejaculatory control; higher sexual satisfaction and lower levels of sexual distress. Although older men may experience PE and perceive less control, they are more likely to be satisfied and experience less distress about PE.

The rates of recorded ED based on diagnosis using the IIEF-5 scores were similar to other studies [32,33,39]. The increase in ED rates with age is also in line with past research. As men age, they are more likely to experience ED. The current findings were confirmed through an analysis of variance where differences were found between age groups [39–41]. Erectile dysfunction was associated with the domains of the IPE. Higher rates of perceived control and satisfaction, and lower distress were correlated with lower levels of erectile dysfunctions.

A relationship exists between the presence of PE and ED as confirmed in this study. Age was found to be associated with each of the variables. Further analysis through partial correlations however found that, when controlled, age had little effect. In exploring the predictability of ED as an outcome of PE, only *control* was found to be a significant predictor, as presented in Table 6. This suggests that when working with men who experience PE, then lower level of perceived control are more likely to indicate the presence of ED.

**Table 6:** Partial correlations between the domains of the IPE and IIEF-5 scores.

IPE Domain	IIEF-5 *rho*	*P* value	Change from zero-order
**Control**	.435	.000	0.033
**Sexual satisfaction**	.353	.000	0.038
**Distress**	.411	.000	0.024

Using Jannini, Lombardo and Lenzi model of PE-ED, men who experience PE and ED are also likely to perceive less control as their focus on controlling ejaculation is likely to result in distraction and erection loss [16]. The loss of erection in turn is requires greater arousal resulting in a rapid ejaculation. This circular relationship is likely to result in perceptions of no control. Further investigation is required to confirm the role of perceived control in predicting PE induced ED.

The main limitation of this study is the focus on Czech nationals. The reliance on self-report measures also leads to possible limitation as identified in this study. These limit the generalizability of the results to wider populations. A further limitation is the application of correlational analysis which does not allow for confirmed causality. Future research can explore the causal relationships between variables.

Subsequent research can also focus on the psychobiological factors common to erectile and ejaculatory functions, given the key point they both have in maintaining a good sexual health, and the high prevalence of ED and PE in society. This link should also be taken into account when developing new lines of therapy to improve both functions.

As has been identified, further research is required in to the relationship between self-report and objective measures of sexual wellbeing. In this study, no relationship was found to exist with less men self-reporting the presence of PE and ED than identified through IELTs (admittedly still self-reported) and application of the IIEF-5 for diagnosing ED. The role of control in predicting ED for PE sufferers also, is recommend for further research. Given the limited research on the role of ED as an outcome of PE, more research into this relationship is warranted generally. Age was found to be associated with all variables yet was found to have limited impact on further analysis. Future research could consider age as a mediator or moderator of the relationship between PE and ED given the role age has in both of these conditions. The mediation-moderation relationship could be explored with the three domains of the IPE and its function between PE and ED.

The obtained results support a positive link between ejaculatory and erection functions [15, 28], Furthermore, when attention is focused on the variable of erectile function, it seems that people with greater erection problems score less on the dimensions of control and satisfaction with erectile ejaculation, and report greater distress. These results coincide with the meta-analysis of Corona et al 2015 [28]. The current results must be interpreted with caution as the authors have not investigated diagnoses, but only the risk of experiencing PE-ED.

The results of this study also match what is frequently observed in clinical psychosexual and sexual medicine practice, where men report erectile function issues preceded by difficulties with the ejaculatory function [16]. Therefore, a set of factors do exist which would affect both functions and explain the PE-ED link. Both functions share biological mechanisms in common [17], whose appropriate functioning would facilitate a proper development of both processes, and would be a key for good sexual health. For example, the use of drugs like sympatholytic, selective serotonin reuptake inhibitors, or methadone, would affect both functions [42]. Endocrine alterations such as hyperthyroidism, or neurologic diseases like diabetic neuropathy, can also cause disturbances in erectile and ejaculatory functions at the same time. Psychological variables such as distress and mood disorders also have a decisive role in both functions [18]. In this context, distress affects both functions by generating an increase in sympathetic tone [43], and this anxiety, in many cases, would be caused by thoughts and concerns about sexual performance, therefore becoming a vicious circle.

Erectile and ejaculatory functions would be affected by sexual performance anxiety [44]. This would cause an increase in sympathetic tone, hindering erection due to the peripheral vasoconstriction it generates, and in turn accelerating the ejaculatory reflex, which would increase the initial distress levels upon confirmation of a poor performance.

The strength of this study is the representativeness of their sample, due to its size and selection criteria, and because the measures used (notwithstanding the limitation on generalizability noted above).

## Conclusions

It appears PE and ED functions are related and that there are biologic and psychological factors common for appropriate maintenance of both. In this context, it is recommended that given this relation, in the clinical management of PE and ED, an evaluation of both functions should be made, regardless that only one of them is the reason for seeking psychosexual and/or medical attention, which would facilitate the selection of a more appropriate treatment, and a better understanding of the underlying mechanisms of these dysfunctions.
